# Elucidation of the genetic determination of clutch traits in Chinese local chickens of the Laiwu Black breed

**DOI:** 10.1186/s12864-023-09798-0

**Published:** 2023-11-16

**Authors:** Jie Wang, Zhansheng Liu, Dingguo Cao, Jie Liu, Fuwei Li, Heguo Han, Haixia Han, Qiuxia Lei, Wei Liu, Dapeng Li, Jianxia Wang, Yan Zhou

**Affiliations:** 1 Poultry Breeding Engineering Technology Center of Shandong Province, Poultry Institute, Shandong Academy of Agricultural Sciences, Jinan, 250023 Shandong China; 2 Shandong Animal Husbandry General Station, Jinan, 250023 China; 3 Lijin County Center for Animal Disease Control, Lijin, 257400 China; 4 Administrative Examination and Approval Service Bureau of Lijin County, Lijin, 257400 China

**Keywords:** Chicken, Genome, GWAS, Laying rate, Clutch traits

## Abstract

**Background:**

Egg laying rate (LR) is associated with a clutch, which is defined as consecutive days of oviposition. The clutch trait can be used as a selection indicator to improve egg production in poultry breeding. However, little is known about the genetic basis of clutch traits. In this study, our aim was to estimate genetic parameters and identify quantitative trait single nucleotide polymorphisms for clutch traits in 399 purebred Laiwu Black chickens (a native Chinese breed) using a genome-wide association study (GWAS).

**Methods:**

In this work, after estimating the genetic parameters of age at first egg, body weight at first egg, LR, longest clutch until 52 week of age, first week when the longest clutch starts, last week when the longest clutch ends, number of clutches, and longest number of days without egg-laying until 52 week of age, we identified single nucleotide polymorphisms (SNPs) and potential candidate genes associated with clutch traits in Laiwu Black chickens. The restricted maximum likelihood method was used to estimate genetic parameters of clutch pattern in 399 Laiwu Black hens, using the GCTA software.

**Results:**

The results showed that SNP-based heritability estimates of clutch traits ranged from 0.06 to 0.59. Genotyping data were obtained from whole genome re-sequencing data. After quality control, a total of 10,810,544 SNPs remained to be analyzed. The GWAS revealed that 421 significant SNPs responsible for clutch traits were scattered on chicken chromosomes 1–14, 17–19, 21–25, 28 and Z. Among the annotated genes, *NELL2*, *SMYD9, SPTLC2*, *SMYD3* and *PLCL1* were the most promising candidates for clutch traits in Laiwu Black chickens.

**Conclusion:**

The findings of this research provide critical insight into the genetic basis of clutch traits. These results contribute to the identification of candidate genes and variants. Genes and SNPs potentially provide new avenues for further research and would help to establish a framework for new methods of genomic prediction, and increase the accuracy of estimated genetic merit for egg production and clutch traits.

**Supplementary Information:**

The online version contains supplementary material available at 10.1186/s12864-023-09798-0.

## Background

Egg laying rate (LR), which equals the number of eggs laid divided by the number of days in the recording period, is the trait with the highest economic weight in breeding programs for laying hens. Egg LR is associated with a clutch, which is defined as consecutive days of oviposition. Successive clutches are separated by one or more days of pause. The length and number of clutches are controlled by the ovulatory cycle directly observed by the oviposition cycle and controlled by the circadian rhythm and the internal cycle of follicular growth and maturation [1, 2]. Egg-laying is characterized by two temporal oviposition traits, namely oviposition time and oviposition interval (*i.e*., the time period between two consecutive ovipositions in the same clutch). Long-term selection has decreased the genetic variability in the egg LR [3]. Since the clutch number and oviposition traits are more basic biological traits than the egg LR, the evaluation of clutch traits can provide further insight into the difference between highly productive birds and inferior layers, compared to a simple total egg number count or rate of laying. As a more basic biological trait, clutch pattern may be more heritable. Studies performed in individual cages have shown that the clutch number, mean oviposition time, and mean oviposition interval were more heritable than the LR and were favorably genetically correlated with the LR [1, 3–7].

Clutch size can be used to describe the individual laying pattern and has been considered as a trait for selection [4, 6, 8, 9]. Early selection methods are commonly employed to improve reproductive performance, using the 300-day egg number as the selection criterion, and sacrificing some accuracy to decrease the generation interval. As an inherent trait of female chickens, clutch traits are also applicable in evaluating reproductive performance. Clutch traits are mainly influenced by breed, and egg-laying chickens have earlier average ovulation times and exhibit better continuous laying performance. Thus, choosing clutch traits as selection indicators offers poultry breeders a promising way to upgrade egg production. Previous studies have shown that selecting for clutch traits can improve egg production in laying hens [4, 6, 9]. Egg production was genetically positively correlated with average (0.57 and 0.61) and maximum clutch size (0.61 and 0.45) and genome-wide association studies (GWAS) in chickens have identified regions related to clutch traits, including the *WASH1, NPVF* and *FOXO3* genes [3, 4, 10, 11]. Despite the increased use of local chicken breeds in recent years, little research has examined the genetic parameters of their laying traits, particularly clutch traits. Laiwu Black chicken is a native Chinese breed and is currently undergoing selective breeding for its egg-laying capacity. According to Chinese consumption habits, eggs produced by local chicken breeds can achieve higher prices compared to imported commercial chicken breeds, sometimes reaching prices that are two to three times higher. Therefore,, we discuss the underlying principles and feasibility of using clutch traits as a breeding index to improve egg production and enhance knowledge on breeding laying hens while promoting the development of local chicken breeds.

Ultimately, we aimed here to estimate the genetic parameters and identify quantitative trait single nucleotide polymorphisms (QTSs) associated with clutch traits in a population of 399 purebred Laiwu Black chickens using the GWAS approach. This study provides novel insights into the genetic architecture underlying chicken clutch traits and is important for the practical implementation of genomics in chicken breeding.

## Material and methods

### Ethics statement

All animal experiments were carried out according to the Directory Proposals on the Ethical Treatment of Experimental Animals, established by the Ministry of Science and Technology (Beijing, China) and were approved by the the Science Research Department of the Shandong Academy of Agricultural Sciences (SAAS) (Jinan, China), (Approval number: SAAS-2019–029, Data:2019–3-18). A total of 399 Laiwu Black chicken were obtained from an experimental farm of the Poultry Institute, Shandong Academy of Agricultural Sciences (PI, SAAS, Jinan, China). All eggs were incubated using a standard procedure and chickens were raised in cages under continuous lighting using standard conditions of temperature, humidity on the PI, SAAS, farm.

### Animals

**Fig. 1:**
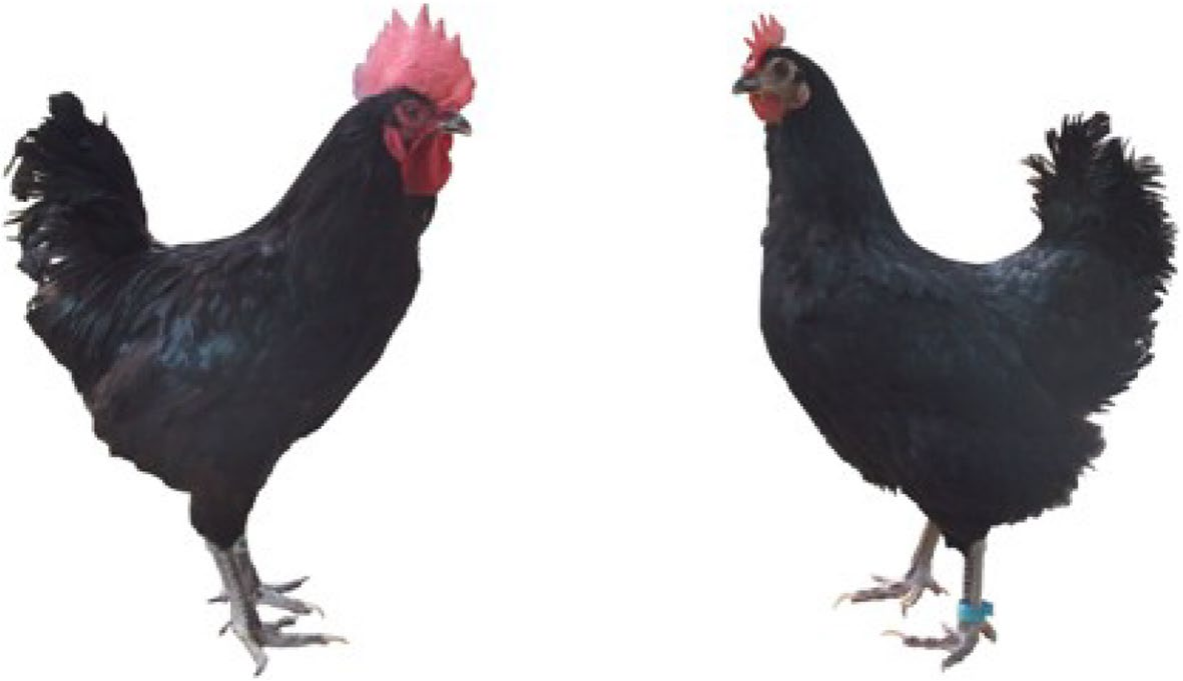
Laiwu Black chickens are a dual-purpose breed for both egg-laying and meat production. The average bodyweight of 8-week-old roosters is 581 g, while the average weight of hens is 512 g. The onset of laying occurs at approximately 145 days, with an average bodyweight of 1,848 g for roosters and 1,399 g for hens. The annual egg production is around 175 eggs

The Laiwu Black chicken (eggs with light brown eggshell) is a slow growing breed and an important source of both meat and eggs (Fig. 1). However, the lack of efficient breeding systems in the purebred lines has resulted in the inefficient application of additive effect of laying traits. Selection for part- or whole-year number of eggs or for LR is a general approach to improve egg production in hens that has contributed to some positive genetic progress. Currently, Laiwu black chickens have an average annual yield of about 175 eggs. The chickens were raised according to the breeding and management procedures of Laiwu black chickens. The experimental chickens were kept in cages throughout the whole process, including 0–7 weeks of age as brooding stage, 8–16 weeks of age as growing stage, and at about 15 weeks of age were transferred to the laying house. The chickens were raised in a single cage, with ad libitum access to food and water, and routine immunization. Chickens were kept under natural light during the growing stage, growing stage, followed by 16 h:8 h light:dark cycle. In the chicken coop, the temperature is controlled by a fan humidification curtain, and the feeding and manure cleaning are mechanized. Within generation records are summarized until the same age for all hatches. All chickens (culled layers) were sold to practitioners who decided to continue feeding or slaughter them after 52 weeks.

### Phenotype

Data were obtained from an egg production line (399 chickens), namely the Laiwu Black breed. Egg number for all individuals was recorded daily from the first egg to 52 weeks and all individual egg production records were recorded independently. Days with two eggs were split into 2 days of one egg if there was no eggs the preceding or subsequent day, otherwise both eggs were counted on the same day. All eggs were counted as parts of clutches, including those with defects. A clutch was defined as the number of eggs laid on consecutive days without a break. Starting with the first egg laid by a hen, the following parameters were calculated: number of clutches (NUMC), longest clutch until 52 week of age (LC), the first week when the longest clutch starts (FWLCS), the last week when the longest clutch ends (LWLCE) and the longest number of days without egg-laying until 52 week of age (LNDWEL). To investigate genetic variance component with standard traits used for selection, the age at first egg (AFE), body weight at first egg (BWFE) and LR after first egg (%; number of eggs to 52 weeks (364 days) of age/(364 − age of the first egg) × 100%) were also included in the analysis.

### Genotype

Whole blood was collected from all 399 Laiwu Black hens by venipuncture at 120 days of age. For all birds, blood was collected from wing veins and rapidly frozen and stored at -20 °C. Total genomic DNA was extracted using the conventional phenol–chloroform method, and the quality and quantity of the DNA were determined using a NanoDrop ND-2000 spectrophotometer (Thermo Fisher Scientific Inc., Waltham, MA, USA) and by agarose gel electrophoresis.

Each DNA sample of adequate quality was used to generate paired-end libraries by standard procedures. All libraries were sequenced on DNBSEQ™ Sequencing System (BGI Genomics, Shenzhen, China). Raw data were processed using SOAPnuke [12] and to ensure the quality of the data used in further analysis low-quality reads and PCR duplications reads were removed. After quality control, the average clean read sequence coverage was 7 × . This depth ensured the accuracy of variant calling and genotyping and met the requirements for population genetic analysis.

The Burrows-Wheeler aligner [13] was used to map clean reads to the chicken reference genome (htttp://ftp.ensembl.org/pub/release-96/fasta/gallus_gallus/dna/). The Samtools v1.2 software [14] was used to sort reads, and the MarkDuplicates program implemented in Picard tools v1.13 (http://broadinstitute.github.io/picard/) was used to remove duplicate reads resulting from polymerase chain reaction (PCR) amplification. Reads mapped to two or more places were likewise screened out. The Genome Analysis Toolkit [15] Haplotype Caller was used for single nucleotide polymorphism (SNP) calling through local re-assembly of haplotypes for the population. SNPs were then screened before further analysis using the GATK Variant Filtration tool with the following settings: QD < 2.0, ReadPosRankSum < -8.0, FS > 60.0, QUAL < 50.0, DP < 200.

### Data quality control and imputation of SNPs

SNPs with an inheritance or genotyping error, a minor allele frequency < 5%, a call rate < 95%, and not assigned to GGA1-GGA28, GGZ and GGW were screened out. Ultimately, after quality control, 10,810,544 SNPs distributed on 30 chicken chromosomes remained. Accordingly, a total of 10,810,544 SNPs and 399 chickens were used for the analysis.

### Principal component analysis

Principal component analysis (PCA) was performed on all SNPs using PLINK (v 1.9) software [16] for the analysis of population structure. Plots of the first, second and third PCs from the PCA were plotted using ggplot2 package in R environment [17].

### Estimation of genetic parameters and genetic correlations

SNP-based heritability (h^2^ SNP) was calculated using the GCTA v1.93.2 beta software [18] based on the genetic relationship matrix (GRM) between pairs of individuals [19]. Univariate and bivariate animal models were fitted by restricted maximum likelihood (REML). The genetic-statistical model was defined as follows:

$${Y}_{i}={X}_{i}{b}_{i}+{Z}_{i}{u}_{i}+{e}_{i}$$

where ${Y}_{i}$ is a vector of clutch traits; ${X}_{i}$ and ${Z}_{i}$ are incidence matrices for ${b}_{i}$ and ${u}_{i},$ respectively; b_i_ is a vector of fixed effect; ${u}_{i}$ is a vector of polygenic effects with a variance–covariance structure of $u\sim N\left(0,G{\sigma }_{u}^{2}\right);$ G is the GRM between individuals; $-{\sigma }_{u}^{2}$ is the polygenic variance; ${e}_{i}$ is a vector of random residual effects with ${e}_{i}\sim N(0,I{\sigma }_{e}^{2})$; *I* is an identity matrix of dimension n × n (with a sample size *n* = 399).

### Genome-wide association studies

Briefly, we started by filtering out SNPs with an inheritance or genotyping error, a minor allele frequency < 5%, and a call rate < 95%. Eventually, after quality control of genotypes, a total of 10,810,544 SNPs and 399 individuals were left for analysis. Linkage disequilibrium (LD) pruning was then performed with a window size of 25 SNPs, a step of five SNPs, and r threshold of 0.2, identifying 1,462,803‬ independent SNPs and LD blocks. The sample structure was visualized by PCA using eigenvalues as coordinates. The Efficient Mixed-Model Association eXpedited (EMMAX) tool was then applied to all informative SNPs using the BN kinship matrix for genome-wide association mapping [20]. The top three principal components contributing to the majority of the population structure variations were included in the model to control the population structure. The EMMAX algorithm is efficient for controlling population stratification, especially the between-generation genotype differences central to the present study [20, 21], considering that population structure helps minimize false positives and increases statistical power. After this analysis, a Manhattan plot was generated from the calculated -log10 (*P*-value) for each SNP. The threshold for genome-wide significance was determined based on 5% Bonferroni correction with the estimated 1,462,803 independent SNPs, giving a value of 0.05/1,462,803‬ = 3.42E-8 [-log10 (*P*-value) = 7.47], while the threshold for suggestive significance was 1/1,462,803 = 6.84E-7 [-log10(*P*-value) = 6.16]. The Manhattan and quantile–quantile (Q-Q) plots for traits were generated using the “CMplot” package in R.

### Phenotypic variation explained

The phenotypic variation explained (PVE) was calculated using the following formula:

$$\mathrm{PVE }= \frac{2{\beta }^{2}MAF(1 - MAF)}{2{\beta }^{2}MAF(1 - MAF) + (se(\beta ){) }^{2}2NMAF(1 - MAF)}$$

where β represents the effect value from the GWAS results, MAF denotes the minimum allele frequency of the SNP, and N represents the number of individuals involved in the GWAS analysis.

### Bioinformatics analysis of candidate genes

Using the VEP software [22] (http://asia.ensembl.org/Tools/VEP), SNPs were assigned to genes if they were within the genomic sequence of a gene or within a flanking region of 5 kb up- or downstream of a gene, to include SNPs located in gene regulatory regions. The Ensembl GRCg6a database was used as a reference (http://asia.ensembl.org/Gallus_gallus/Info/Index).We then identified the biological functions of these genes in PubMed (https://pubmed.ncbi.nlm.nih.gov). To elucidate the biological functions of the genes, we performed the Kyoto Encyclopedia of Genes and Genomes (KEGG) pathway and gene ontology (GO) enrichment analysis using the KOBAS 3.0 online platform [23]. The significance of the pathways enriched in genes was calculated considering a *P*-value of < 0.05 as significant. The simplify enrichment analysis of GO terms were generated using the “simplifyEnrichment” package in R environment [24].

## Results

### Summary of sequencing and genome variants

In this study, we whole-genome re-sequenced 399 chicken samples. A total of 2.14 Tb of high-quality sequencing data was aligned against the reference chicken genome (GRCg6a) (Table S1). The average mapping rate for all individuals was approximately 99.17%, the average coverage (> = 1 ×) for all individuals was approximately 94.64%, and the average depth was approximately 7.41 × (Table S2). Following the screening criteria listed in the “Material and methods” section, a total of 10,810,544 genome-wide population SNPs were obtained (Table S3).

### Phenotype and genetic parameter statistics

Descriptive statistics of the AFE and clutch traits across the entire egg-laying period are shown in Table 1. The mean value of the AFE in this population was 138 days, which meant that hens started laying eggs at about 20 weeks of age. The SNP-based heritability estimate (Table 2) was high for the AFE (0.76 ± 0.16) and LC trait (0.59 ± 0.16), but relatively low (0.06) to moderate (0.36) for other clutch traits. In this population, the LR trait at 52 weeks showed moderate heritability (0.42) and a strong genetic positive correlation (0.796) with LC. In contrast, it showed a negative correlation with NUMC and LNDWEL (-0.688, -0.984) (Table S4).

**Table 1:** Descriptive statistics for the AFE, BWFE and clutch traits

Trait	N	Mean	SD	Max	Min
AFE (d)	397	138.19	10.41	171	104
BWFE (g)	397	1440.57	136.36	2177	1037
LR (%)	344	68.16	11.73	94.71	21.83
LC (d)	395	13.01	10.39	75	2
FWLCS (week)	395	24.49	4.67	42	17
LWLCE (week)	395	26.12	4.66	43	18
NUMC (n)	395	33.51	10.21	56	2
LNDWEL (d)	395	11.27	16.46	136	1

*Abbreviations*: *AFE* Age at first egg, *BWFE* Body weight at first egg, *LR* Laying rate, *LC* Longest clutch until 52 week of age, *FWLCS* The first week when the longest clutch starts, *LWLCE* The last week when the longest clutch ends, *NUMC* Number of clutches, *LNDWEL* The longest day with no egg laid until 52 week of age, *N* Number of animals, *SD* Standard deviation, *Max* Maximum, *Min* Minimum

**Table 2:** SNP-based heritability estimates for clutch traits

Trait	V_g_	V_e_	V_p_	h^2^
AFE	74.09 ± 17.6	23.64 ± 15.42	97.73 ± 7.14	0.76 ± 0.16
BWFE	6117.72 ± 2636.70	11754.48 ± 2720.40	17872.2 ± 1278.28	0.34 ± 0.15
LR	54.31 ± 21.62	76.05 ± 21.93	130.37 ± 9.95	0.42 ± 0.16
LC	56.95 ± 16.77	38.98 ± 15.58	95.93 ± 6.94	0.59 ± 0.16
FWLCS	3.37 ± 2.86	18.27 ± 3.26	21.64 ± 1.56	0.16 ± 0.13
LWLCE	1.33 ± 2.36	20.3 ± 2.94	21.64 ± 1.56	0.06 ± 0.11
NUMC	34.79 ± 13.38	61.16 ± 13.74	95.95 ± 6.88	0.36 ± 0.14
LNDWEL	50.25 ± 32.7	216.28 ± 37.11	266.53 ± 19.15	0.19 ± 0.12

*Abbreviations*: *AFE* Age at first egg, *BWFE* Body weight at first egg, *LR* Laying rate, *LC* Longest clutch until 52 week of age, *FWLCS* The first week when the longest clutch starts, *LWLCE* The last week when the longest clutch ends, *NUMC* Number of clutches, *LNDWEL* The longest day with no egg laid until 52 week of age. Vg: the variance of additive genetic effects. Ve: the variance of residuals. Vp: the variance of phenotype, Ve = Vg + Ve. h2: heritability

### Genome-wide association study

Association tests for the AFE, BWFE and clutch traits were performed using a univariate linear mixed model. The results of the population structural analysis are shown as PCA plots (Figure S2), revealing that the population of 399 individuals is greatly stratified. The top three principal components were included in the model to control the population structure. A total of 421 unique candidate SNPs (*P* value < 6.84E-07) located on 24 chromosomes were identified (Tables S5 and S6). According to gene functions, 36 phenotype-related SNPs were selected from all the significant SNPs and were annotated in detail.

**Fig. 2:**
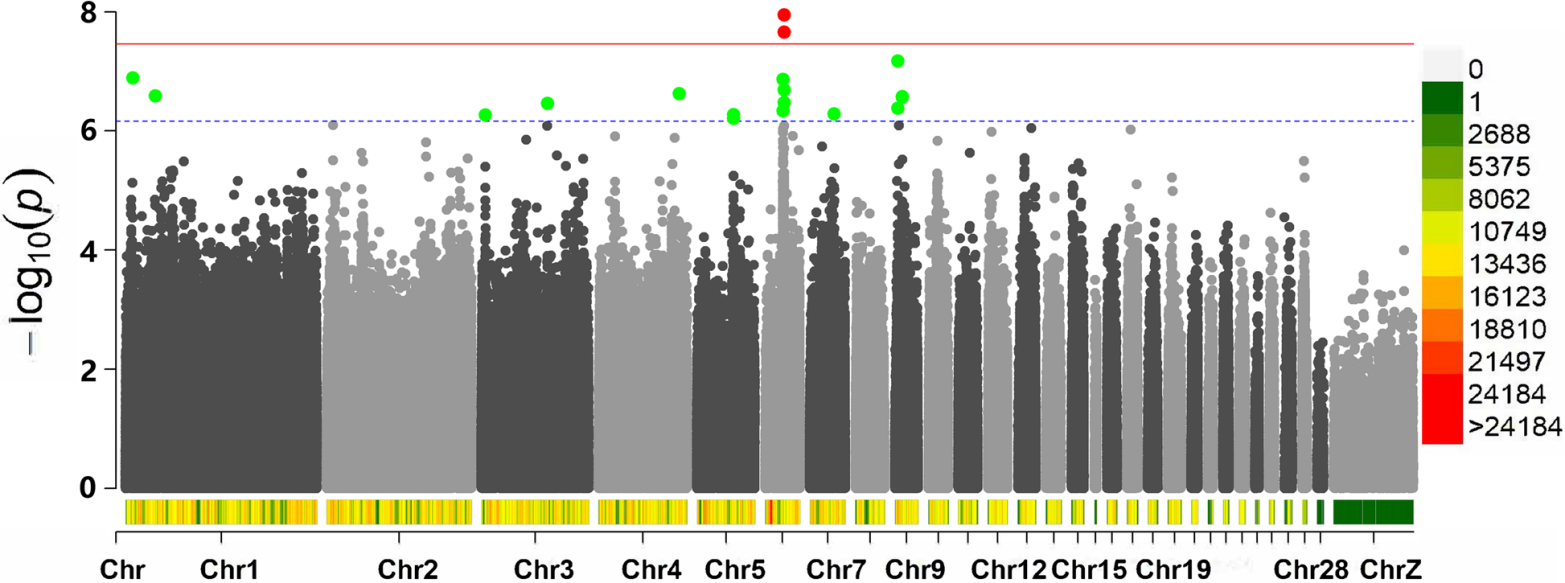
Manhattan plot derived from GWAS for the AFE. Each dot on this figure corresponds to a SNP within the dataset, and the horizontal red and blue lines denote the genome-wide significance (3.42E-8) and suggestive significance thresholds (6.84E-7), respectively. The Manhattan plot contains -log10 observed *P*-values for genome-wide SNPs (y-axis) plotted against their corresponding position on each chromosome (x-axis). The horizontal axis represents the chromosome length in Mb. Different colors correspond to SNP density

**Fig. 3:**
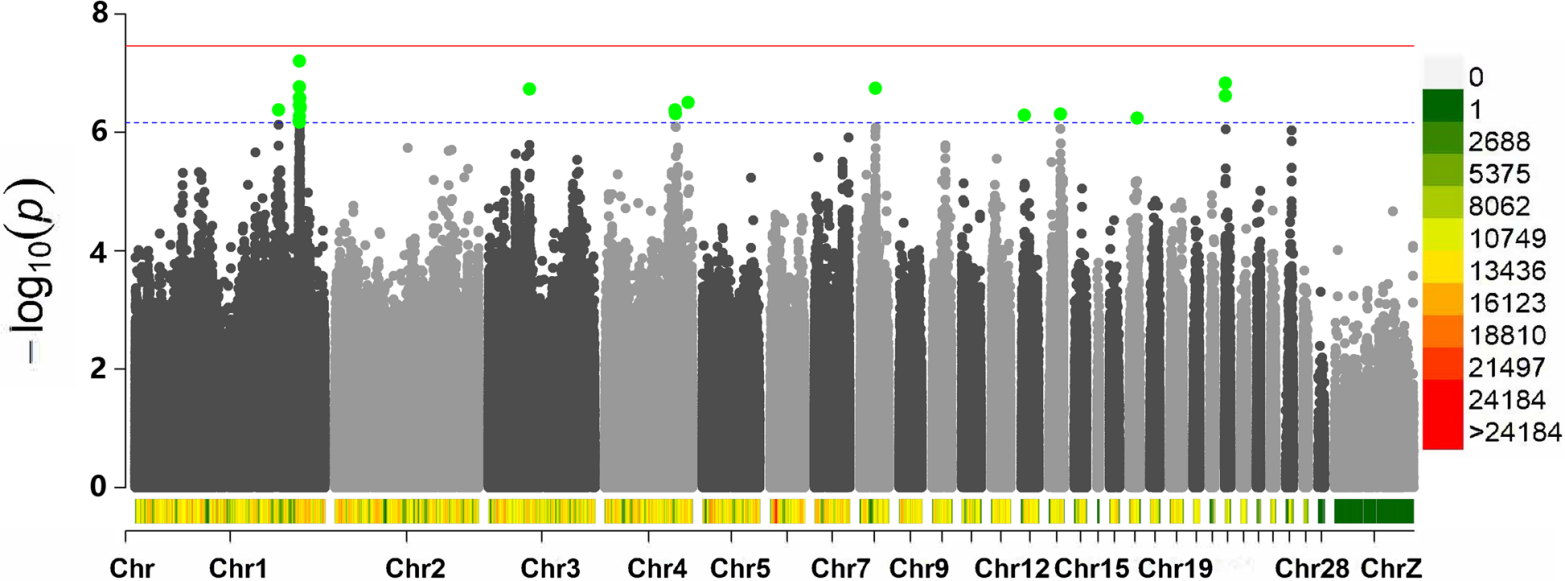
Manhattan plot derived from GWAS for the BWFE. Each dot on this figure corresponds to a SNP within the dataset, and the horizontal red and blue lines denote the genome-wide significance (3.42E-8) and suggestive significance thresholds (6.84E-7), respectively. The Manhattan plot contains -log10 observed *P*-values for genome-wide SNPs (y-axis) plotted against their corresponding position on each chromosome (x-axis). The horizontal axis represents the chromosome length in Mb. Different colors correspond to SNP density

**Fig. 4:**
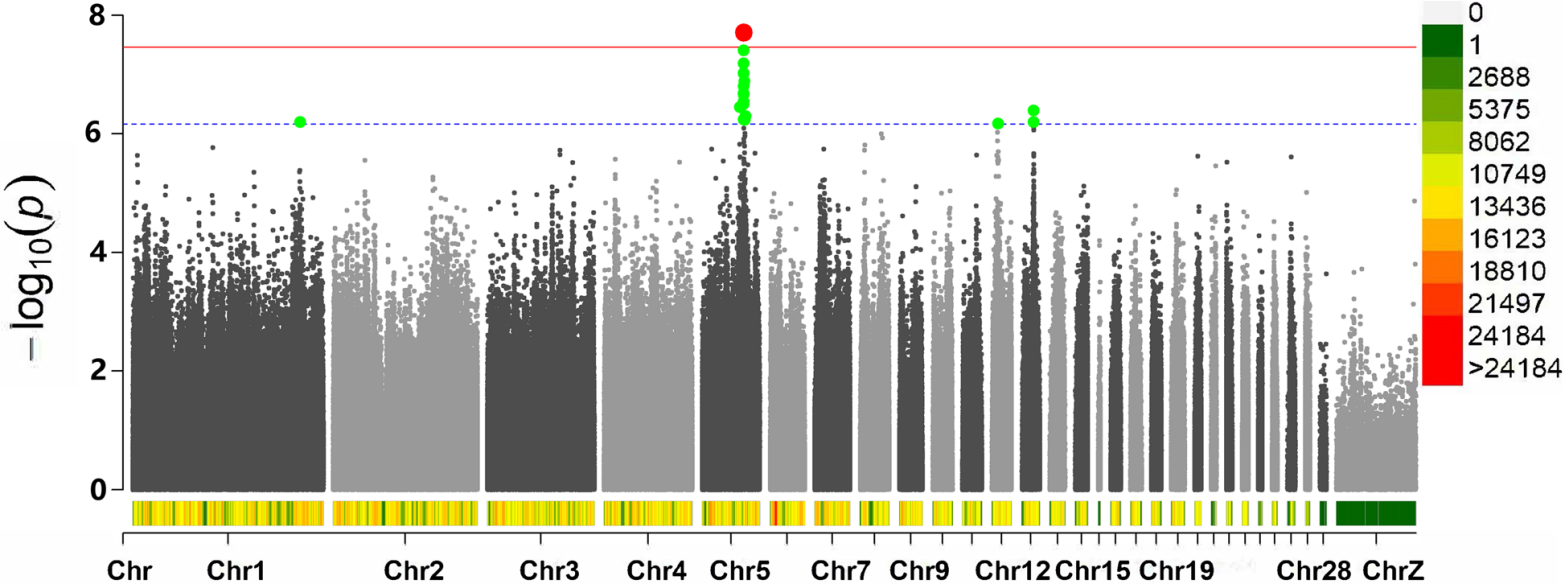
Manhattan plot derived from GWASs for the LR. Each dot on this figure corresponds to a SNP within the dataset, and the horizontal red and blue lines denote the genome-wide significance (3.42E-8) and suggestive significance thresholds (6.84E-7), respectively. The Manhattan plot contains -log10 observed *P*-values for genome-wide SNPs (y-axis) plotted against their corresponding position on each chromosome (x-axis). The horizontal axis represents the chromosome length in Mb. Different colors correspond to SNP density

**Fig. 5:**
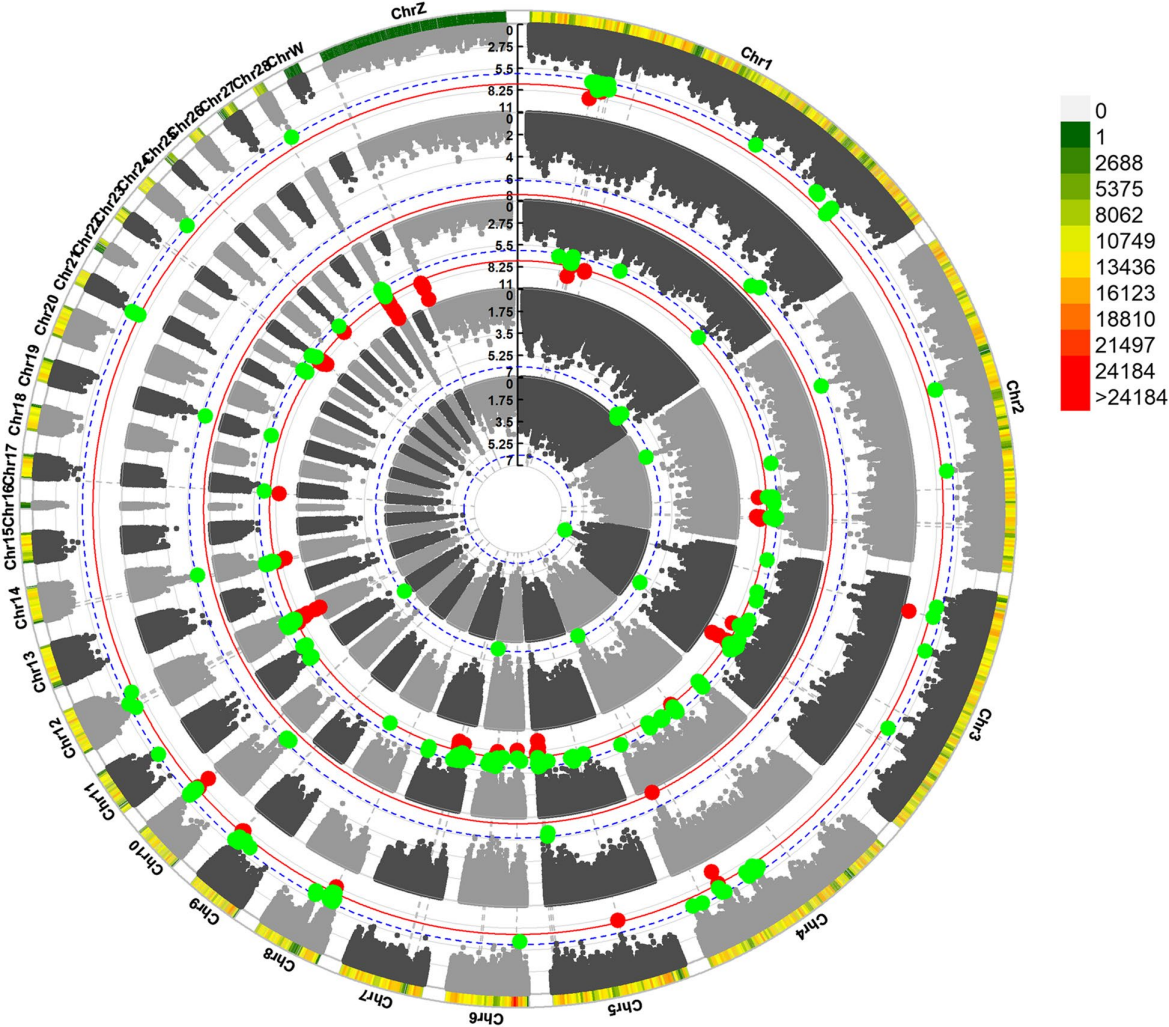
Circle-Manhattan plot derived from GWAS for clutch traits. Each dot on this figure corresponds to a SNP within the dataset, and the horizontal red and blue lines denote the genome-wide significance (3.42E-8) and suggestive significance thresholds (6.84E-7), respectively. The Manhattan plot contains -log10 observed *P*-values for genome-wide SNPs (y-axis) plotted against their corresponding position on each chromosome (x-axis). The horizontal axis represents the chromosome length in Mb. Different colors correspond to SNP density. The circle from outside to inside are LC, FWLCS, LNDWEL, LWLCE, NUMC traits

Five of them scattered on two different chromosomes were associated with the AFE (Table 3), five of them scattered on three different chromosomes were associated with BWFE (Table 4), two of them scattered on two different chromosomes were associated with the LR (Table 5), and the remaining 24 significant SNPs were related to clutch traits (Table 6). The Manhattan plots for the AFE, BWFE, LR and clutch traits are shown in Figs. 2, 3, 4 and 5, respectively. The quartile-quartile (QQ) plots for the AFE, BWFE, LR and clutch traits are shown in Figure S3. The linking results of particular individual SNP genotypes to any particular individual phenotype trait are shown in Table S7.

**Table 3:** GWAS and annotations of suggestive SNPs for the AFE

Chr	BP	ALT/REF	Beta	*P* value	Consequence	Candidate/Nearest gene	PVE
1	7413930	C/T	-8.50	1.3E-07	intron	*FRMD4A*	6.79%
1	30524767	A/G	8.98	2.6E-07	intron	*NELL2*	5.54%
6	19003506	T/A	-4.10	4.6E-07	intron	*PTPN20*	5.80%
6	19039248	A/G	-3.86	1.4E-07	intron	*MAPK8*	6.18%
6	19039251	T/C	-3.81	1.4E-07	intron	*MAPK8*	6.21%

*Abbreviations*: *ALT/REF* Alternative vs reference allele, *Chr* Chromosome, *BP* Physical position, *Beta* The estimated coefficient

**Table 4:** GWAS and annotations of suggestive SNPs for the BWFE

CHR	BP	ALT/REF	Beta	*P *value	Consequence	Candidate/Nearest gene	PVE
1	149332781	G/A	-53.86	4.2E-07	intron	*GPC5*	6.49%
1	171732987	G/A	64.88	3.9E-07	intron	*CKAP2*	6.03%
					upstream	*VPS36*	
1	171732996	G/C	63.62	3.7E-07	intron	*CKAP2*	6.06%
					upstream	*VPS36*	
8	16338421	A/T	69.53	1.8E-07	intron	*SH3GLB1*	6.25%
18	7998007	T/G	-100.42	5.7E-07	intron	*MAP2K6*	6.04%

*Abbreviations*: *ALT/REF* Alternative vs reference allele, *Chr* Chromosome, *BP* Physical position, *Beta* The estimate coefficient

**Table 5:** GWAS and annotations of suggestive SNPs for the LR

Chr	BP	ALT/REF	Beta	*P* value	Consequence	Candidate/Nearest gene	PVE
1	173782660	A/G	-5.12	6.4E-07	downstream	*SMAD9*	6.07%
5	39490051	G/T	-5.79	3.6E-07	intron	*SPTLC2*	6.33%

*Abbreviations*: *ALT/REF* Alternative vs reference allele, *Chr* Chromosome, *BP* Physical position, *Beta* The estimate coefficient

**Table 6:** GWAS and annotations of suggestive SNPs for the clutch traits

Trait	CHR	BP	ALT/REF	Bata	*P* value	Consequence	Candidate/Nearest gene	PVE
LC	1	35178616	G/A	-8.18	7.5E-08	upstream	*IFNG*	7.82%
LC	1	40548144	C/T	-7.04	1.1E-07	intron	*PPFIA2*	6.81%
LC	3	16867987	C/G	-5.92	1.1E-07	intron	*GPCPD1*	7.09%
LC	3	34132127	C/T	-7.01	2.0E-07	intron	*SMYD3*	7.10%
LC	4	50279449	T/C	-7.39	1.7E-07	intron	*SLC4A4*	6.12%
LC	4	56572493	A/T	-5.96	4.5E-07	intron	*CAMK2D*	6.49%
LC	4	56956942	G/A	-7.89	9.8E-08	intron	*ANK2*	7.52%
LC	8	6861178	A/G	-9.73	6.4E-09	intron	*RASAL2*	9.04%
LC	9	13586651	A/G	-7.34	2.1E-07	intron	*FGF12*	6.89%
LC	9	19708862	A/G	-7.79	2.3E-07	intron	*PLD1*	6.86%
LC	10	18641056	C/A	-7.44	4.7E-10	intron	*MEGF11*	9.32%
LC	10	18921,595	A/G	-6.12	9.4E-08	intron	*MAP2K1*	6.87%
LC	12	15204,994	T/C	-7.71	6.8E-08	intron	*SUCLG2*	7.61%
FWLCS	1	167312472	A/G	-2.26	2.0E-07	intron	*VWA8*	7.06%
FWLCS	4	82173143	T/C	-1.86	2.9E-08	intron	*HTT*	7.88%
FWLCS	10	2038843	A/G	-1.72	3.0E-07	intron	*PKLR*	6.45%
FWLCS	14	6153293	T/C	-2.23	4.7E-07	upstream	*SOX8*	6.09%
LWLCE	1	167312472	A/G	-2.21	2.5E-07	intron	*VWA8*	6.59%
LWLCE	4	82097182	A/C	-2.39	4.8E-07	upstream	*MSANTD1*	5.62%
LNDWEL	1	39566858	C/T	-14.198	1.5E-09	intron	*PAWR*	8.72%
LNDWEL	3	82310311	C/T	-17.162	1.3E-09	intron	*ASRGL1*	10.09%
LNDWEL	7	10368909	C/T	-14.099	7.1E-09	upstream	*PLCL1*	8.12%
LNDWEL	12	7864695	C/T	-13.236	2.2E-08	intron	*CACNA2D3*	7.51%
LNDWEL	Z	1719462	G/A	-11.475	8.4E-11	upstream	*SKOR2*	10.91%

*Abbreviations*: *ALT/REF* Alternative vs reference allele, *Chr* Chromosome, *BP* Physical position, *Beta* The estimated coefficient, *LC* Longest clutch until 52 week of age, *FWLCS* The first week when the longest clutch starts, *LWLCE* The last week when the longest clutch ends, *NUMC* Number of clutches, *LNDWEL* The longest day with no egg laid until 52 week of age

### Annotation of candidate SNPs

The candidate genes associated with the AFE are listed in Table 3, including FERM domain containing 4A (*FRMD4A*), neural EGFL like 2 (*NELL2*), protein tyrosine phosphatase non-receptor type 20 (*PTPN20*), and mitogen-Activated protein kinase 8 (*MAPK8*). In addition, the candidate genes associated with the BWFE are listed in Table 4, including glypican 5 (*GPC5*), cytoskeleton associated protein 2 (*CKAP2*), vacuolar protein sorting 36 homolog (*VPS36*), SH3 domain containing GRB2 like, endophilin B1 (*SH3GLB1*), and mitogen-activated protein kinase kinase 6 (*MAP2K6*).

The candidate genes associated with the LR are listed in Table 5, including SMAD family member 9 (*SMAD9*) and serine palmitoyltransferase long chain base subunit 2 (*SPTLC2*). Annotations of suggestive SNPs for clutch traits are shown in Table 6. A total of 355 genes containing significant SNPs, and 24 candidate genes associated with clutch traits according to their functions were identified. Some genes, such as glycerophosphocholine phosphodiesterase 1 (*GPCPD1*), SET and MYND domain containing 3 (*SMYD3*), solute carrier family 4 member 3 (*SLC4A4*), fibroblast growth factor 12 (*FGF12*), phospholipase D1 (*PLD1*), mitogen-activated protein kinase kinase 1 (*MAP2K1*), von Willebrand factor A domain containing 8 (*VWA8*), Myb/SANT DNA binding domain containing 1 (*MSANTD1*), huntingtin (*HTT*), pro-apoptotic WT1 regulator (*PAWR*), asparaginase like 1 (*ASRGL1*), phospholipase C like 1 (*PLCL1*), calcium voltage-gated channel auxiliary subunit alpha2delta 3 (*CACNA2D3*), and SKI family transcriptional corepressor 2 (*SKOR2*), which have been shown to be related to egg number, egg production, litter size, or reproductive traits, are worth a deeper exploration.

### Signaling pathways and gene ontology terms associated with annotated genes

**Fig. 6:**
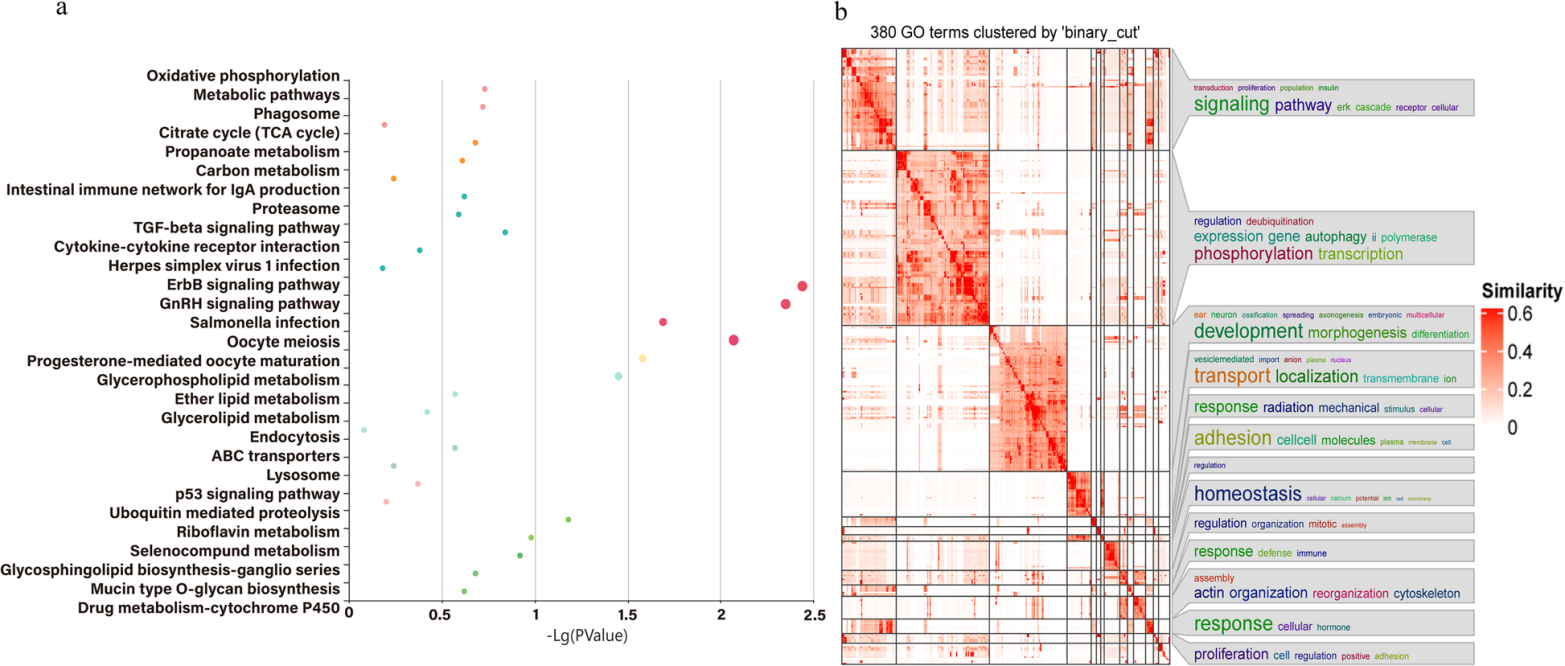
Kyoto Encyclopedia of Genes and Genomes (KEGG) pathway and GO enrichment analysis

Using VEP tools, 140 genes were annotated through 421 unique candidate SNPs, then the Kyoto Encyclopedia of Genes and Genomes (KEGG) pathway and GO enrichment analysis was performed (Tables S8 and S9). As expected, among these candidate gene-enriched pathways, some reproduction-related pathways were identified, including the gonadotropin-releasing hormone (GNRH) signaling pathway, ErbB signaling pathway and Oocyte meiosis, etc., as shown in Fig. 6a. Functional enrichment analysis or gene set enrichment analysis is a basic bioinformatics method that evaluates the biological importance of a list of genes of interest. The GO terms simplifyEnrichment results showed that the list of candidate genes was enriched in terms, such as development, transport, homeostasis and adhesion (Fig. 6b).

## Discussion

Clutch traits are one of the important reproductive performance indicators of hens, which are affected by follicle development. The growth of follicles is affected by factors, such as species, age, environment, hormone levels, nutrition, etc. When ovulation is interrupted for a day or a few days, hens experience intermittent egg-laying. Related research shows that clutch size can be used to describe the individual laying pattern and has been considered as a trait for selection, and clutch trait used as selection indicator of breeding can effectively extend the clutch length, reduce the intermission clutch, and improve egg production [4, 8]. However, to date, the genetic architectures underlying chicken clutch traits are not completely known. Through the estimation of genetic parameters and a GWAS, this study preliminarily revealed the genetic architectures of clutch traits in chickens. Our results provide valuable genomic information to understand the molecular genetic mechanisms of clutch traits of the Chinese native egg-laying chicken breed Laiwu Black.

### Estimates of genetic parameters

Estimates of genetic parameters for clutch related traits revealed that the AFE had the highest heritability estimate (0.76). In the literature, the AFE has been reported as a trait with moderate heritability (0.36–0.55) [4, 25–27], which is not in agreement with our estimates. Our results also showed that the number of clutches had a moderate heritability estimate (0.36), which is similar to related studies showing that the heritability of the number of clutches and average clutch length is around 0.15–0.68 [3, 9, 28, 29].

### Genome-wide association study

Population stratification is a critical issue that can lead to false positive results in GWAS of complex traits. Previous studies have mainly used genomic control, stratification analysis, PCA, and mixed linear model (MLM) association to address this issue. However, in recent years, genomic control and stratification analysis have been less frequently used, and most studies have instead opted for PCA and MLM association for population stratification analysis. For instance, Tang et al. [30] used PCA to address the effect of population stratification on association analysis. Meanwhile, Uzzaman et al. [31] used MLM association to account for population structure in an association analysis of reproductive traits in Yorkshire pigs. In our study, the PCA plots revealed that there was a significant genomic variation among this sample population of 399 individuals of the Laiwu Black breed. We used PCA to assess population stratification and incorporated the first three principal components into the MLM for association analysis to reduce the likelihood of false positive results. Our QQ plots for various trait data revealed the absence of any population stratification phenomenon in the adjusted experimental sample data, and the inflation factors were all under 1.02. Therefore, the association results of our study based on MLM analysis are reliable for candidate gene mining and validation.

In our study, we calculated the PVE for QTS candidates, and some SNPs had PVE results exceeding 10%. Noteworthy, due to linkage disequilibrium (LD) between significant SNPs, the PVE method results in a sum of PVE values being significantly greater than 1 for all SNPs. Given that each SNP represents a gene, a high LD implies that the gene is represented multiple times, leading to an overestimation of the PVE. In theory, the upper limit of the PVE is the heritability (h2). Additionally, PVE values obtained using different models, such as the generalized linear model (GLM) and MLM, differ, indicating that the PVE values obtained in our study for each SNP are only relevant when compared to QTSs with the same phenotype.

### Candidate genes of AFE and BWFE traits

The AFE of chicken is an indicator that is related to reproduction and growth [32]. There is a negative correlation between the AFE (sexual maturity) and the number of eggs produced, that is, the earlier the AFE, the more eggs are laid. However, if hens start laying eggs early when they are underweight, it will have a very negative impact on egg production in the later laying cycles [26, 33–35]. In this study, the *NELL2* and *MAPK8* genes were associated with the AFE of the chicken. Some genes regulate hormone levels, including GNRH, oxytocin (OXT), growth hormone (GH), and thyroid hormone (TH). All these hormones play a crucial role in the female reproductive system [36–41]. NELL2 not only affects the synthesis and secretion of GNRH [42], but is also involved in maintaining the normal female reproductive cycle of mammals [43]. Pulsatile GNRH regulates the expression of gonadotropin subunit genes in a differential manner, with faster frequencies favoring *Lhb* expression and slower frequencies favoring *Fshb* expression. The GNRH-induced *Egr1* gene expression is mediated by MAPK8/9 and MAPK1/3, and both are critical for *Lhb* gene transcription [44]. The internal regulation axis (*NELL2*-GNRH-*MAPK8* axis) regulates the AFE trait of chicken. The *CKAP2* and MAP2K6 genes have been associated with the BWFE. *CKAP2* encodes cytoskeleton associated protein 2, which is involved in cell proliferation and cell survival [45], and is essential for maintaining genomic stability [46]. A previous study showed that the oviduct mass remains unchanged during the laying period in zebra finches [47], and another study suggested that *CKAP2* may participate in the hyperplasia and hypertrophy of the oviduct before the laying of the first egg [48]. MAP2K6 promotes adipogenesis through the regulation of peroxisome proliferator-activated receptor gamma (PPARG) and CCAAT/enhancer-binding protein beta (CEBPB) [49].

### Genes associated with LR and clutch traits

The LR is a trait with high economic value in breeding programs for laying hens, as it reflects the number of eggs laid during days of oviposition. In this study, the *SMAD9* and *SPTLC2* genes were identified as candidate genes associated with the LR trait.

Follicular development and selection are critical factors that affect egg production in poultry. According to recent studies, *SMAD9*, an essential transcription factor in the BMP/SMAD pathway, plays a crucial role in follicular initiation, suggesting the involvement of *SMAD9* in both avian follicular development and ovulation processes [50]. Efficient and healthy development of ovarian follicles is crucial for high laying performance in chickens. Moreover, the rapid growth of oocytes is the result of abundant yolk deposition through blood circulation interactions between somatic and germ cells in the ovary. Vital components of the yolk mass include very low-density lipoprotein (VLDL) and vitellogenin that mature oocytes take up, via VLDL receptor-mediated endocytosis, from blood capillaries located within the Theca layer and gaps between granulosa cells [51]. Research on mice has shown that adenovirus AdSptlc2 (an adenovirus containing *SPTLC2*) infection leads to the downregulation of gluconeogenic genes, resulting in a decrease in plasma glucose levels. Conversely, the same infection leads to increased VLDL secretion, which in turn causes elevated plasma triglyceride levels [52].

As the main factor affecting the reproductive performance of birds, a lighting regime has been applied to modern poultry production. By adopting a suitable light regime, producers can make the start of production of poultry earlier or later, thereby adjusting the egg production rate and laying time. Hens have regular laying intervals under continuous light conditions, have high production efficiency for a certain period of time, and show no laying interruptions. In this study, the *ASRGL1* gene was identified as a candidate gene associated with clutch traits. ASRGL1 is a ubiquitously-expressed asparaginase present in the mammalian uterus and is highly expressed in the brain and testes [53, 54]. *ASRGL1* knockout mice exhibited impaired visual function and showed degeneration and apoptosis of photoreceptor cells [55]. This gene may affect the visual function of chickens and thus affect the clutch traits of chickens.

The change of the clutch cycle Is not only directly affected by the external environmental photoperiod and temperature, but the nutrient intake also affects the clutch cycle change. Delayed ovulation time may be affected by the different ways in which laying hens metabolize material and energy throughout the day. Hens mainly focus on energy metabolism during the day and protein synthesis at night, and thus hens need a sufficient amount of protein throughout the day for optimal egg-laying performance [56]. The *MEGF11* and *SUCLG2* genes were identified as LC-related genes. According the GWAS and functional annotations, the *MEGF11* gene is associated with feed efficiency [57]. and the *SUCLG2* gene is involved in the propanoate metabolic pathway and has been significantly associated with growth in pigs [58]. Moreover, the latter study suggested that *SUCLG2* plays a key role in the regulation of *POU1F1*, which is known to be involved in the expression of growth hormone and prolactin genes [59, 60].

The reproduction of birds is regulated by a series of complex internal and external environments. Seasons, lighting, and environmental stress all have an impact on their reproduction. The internal reproductive Hypothalamic-Pituitary–Gonadal (HPG) axis regulates their reproductive cycle. A related study found that prolactin (PRL) levels in hens were low in the early part of the clutch cycle, while PRL levels increased in the later part of the cycle. PRL levels were inversely correlated with clutch cycle [61]. In general, 4 h before ovulation is the peak of luteinizing hormone (LH) secretion, and the time of LH release of different breeds leads to different ovulation times. Injecting progesterone to hens can also accelerate the ovulation process, indicating that the occurrence and change of endocrine regulation can affect the performance of continuous production [62]. In this study, some genes such as *SMYD3*, *PAWR*, *PLCL1* have been shown to be related to the HPG axis. *SMYD3* is a SET domain-containing protein with histone methyltransferase activity on histone H3-K4. RNA interference-directed down-regulation of *SMYD3* revealed that *SMYD3* is required for endoplasmic reticulum-regulated gene transcription in the estrogen signaling pathway [63]. It has been reported that the pro-apoptotic protein PAWR regulates apoptosis in rat follicles, and follicle-stimulating hormone (FSH) acts by suppressing PAWR expression in the ovary by activating the PKCζ-dependent anti-apoptotic pathway [64, 65]. The phospholipase C-like proteins PLCL1 and PLCL2 are thought to act as sequesters of inositol triphosphates and reduce the signal to release calcium from the intracellular calcium storage [66]. In female reproduction in mice, loss of both *Plcl1* and *Plcl2* results in subfertility, abnormal estrous cycle, and smaller uteri [67]. *Plcl1* and *Plcl2* double-knockout mice apparently grew normally and became fertile, but mutant female mice had an apparently smaller uterus by gross anatomical observation and had more estrous days during the cycles. They also measured serum levels of LH and FSH for 5–6 consecutive days and found them to be significantly higher in the mutant [67].

### Signaling pathways associated with the clutch traits

The regulation of the clutch traits may be mediated through complex interactions among multiple pathways. According to the KEGG pathway enrichment analysis, several well-known pathways associated with reproduction were found to be enriched, including GNRH signaling pathway, ErbB signaling pathway and Oocyte meiosis pathway, etc. GNRH, a hypothalamic neuropeptide, is a main regulator of male and female reproductive function, which is required for reproduction. GNRH is secreted from the hypothalamus and acts on G-protein coupled receptors in the anterior pituitary. GNRH regulates the production and release of LH and FSH gonadotropins, controlling gametogenesis and steroidogenesis [68, 69]. The ErbB receptors signal through Akt, MAPK, and many other pathways to regulate cell proliferation, migration, differentiation, apoptosis, and cell motility. Related studies have found a crosstalk mechanism between GH and PRL and ErbB receptor family signaling [70]. Meiosis is one of the defining events in gametogenesis. Male and female germ cells both undergo one round of meiotic cell division during their development in order to reduce the ploidy of the gametes, and thereby maintain the ploidy of the species after fertilization [71].

Previous studies have identified numerous QTLs associated with AFE and clutch traits. For instance, a GWAS revealed seven and twelve regions linked to egg production in the Rhode Island Red (RIR) and White Leghorn (WL) lines, respectively, with *WASH1*, *NPVF*, and *FOXO3* as candidate genes [72]. However, this study did not produce any overlapping results. The transcriptome results suggest that PRL specifically contributes to the number of eggs at 50 weeks of age or the LR after the first egg, by decreasing oviposition lag within the clutch (*P* < 0.05) and/or increasing the average clutch length (*P* < 0.05) [73]. This finding supports the results of our pathway analysis. In addition, the ErbB pathway may impact the clutch trait via PRL.

## Conclusion

We performed genetic parameter estimation and a GWAS in a Chinese native chicken population to examine genetic architectures underlying clutch traits. The clutch traits showed moderate to high heritability. The *NELL2* gene may affect the AFE through the NELL2-GNRH-MAPK8 axis in chickens. Moreover, the *SMYD9, SPTLC2*, *SMYD3* and *PLCL1* genes, which were identified by annotating genome-wide significant SNPs, can be considered as candidates genes associated with LR and clutch traits. The identified candidate genes and SNPs provide new avenues for further research. Further validation of SNPs and genes identified in this study in a larger cohort would help to establish a framework in methods of genomic prediction, increasing the accuracy of estimated genetic merit for egg production and clutch traits and helping the chicken breeding industry.

### Supplementary Information

**Additional file 1: Figure S1.** SNP density plot across the 30 chromosomes of chicken showing the number of SNPs within a 1-Mb window size. The horizontal axis represents the chromosome length in Mb. Different colors correspond to SNP density. **Figure S2.** Principal component analysis (PCA). **Figure S3.** Q-Q plot derived from GWASs for clutch traits. The Q-Q plot contains expected -log10-transformed *P*-values plotted against the observed -log10-transformed *P*-values. **Figure S4.** Manhattan plot derived from GWASs for the LC. Each dot on this figure corresponds to a SNP within the dataset, and the horizontal red and blue lines denote the genome-wide significance (3.42E-8) and suggestive significance thresholds (6.84E-7), respectively. The Manhattan plot contains -log10 observed *P*-values for genome-wide SNPs (y-axis) plotted against their corresponding position on each chromosome (x-axis). The horizontal axis represents the chromosome length in Mb. Different colors correspond to SNP density. **Figure S5.** Manhattan plot derived from GWASs for the FWLCS. Each dot on this figure corresponds to a SNP within the dataset, and the horizontal red and blue lines denote the genome-wide significance (3.42E-8) and suggestive significance thresholds (6.84E-7), respectively. The Manhattan plot contains -log10 observed *P*-values for genome-wide SNPs (y-axis) plotted against their corresponding position on each chromosome (x-axis). The horizontal axis represents the chromosome length in Mb. Different colors correspond to SNP density. **Figure S6.** Manhattan plot derived from GWASs for the LWLCE. Each dot on this figure corresponds to a SNP within the dataset, and the horizontal red and blue lines denote the genome-wide significance (3.42E-8) and suggestive significance thresholds (6.84E-7), respectively. The Manhattan plot contains -log10 observed *P*-values for genome-wide SNPs (y-axis) plotted against their corresponding position on each chromosome (x-axis). The horizontal axis represents the chromosome length in Mb. Different colors correspond to SNP density. **Figure S7.** Manhattan plot derived from GWASs for the NUMC. Each dot on this figure corresponds to a SNP within the dataset, and the horizontal red and blue lines denote the genome-wide significance (3.42E-8) and suggestive significance thresholds (6.84E-7), respectively. The Manhattan plot contains -log10 observed *P*-values for genome-wide SNPs (y-axis) plotted against their corresponding position on each chromosome (x-axis). The horizontal axis represents the chromosome length in Mb. Different colors correspond to SNP density. **Figure S8.** Manhattan plot derived from GWASs for the LNDWEL. Each dot on this figure corresponds to a SNP within the dataset, and the horizontal red and blue lines denote the genome-wide significance (3.42E-8) and suggestive significance thresholds (6.84E-7), respectively. The Manhattan plot contains -log10 observed *P*-values for genome-wide SNPs (y-axis) plotted against their corresponding position on each chromosome (x-axis). The horizontal axis represents the chromosome length in Mb. Different colors correspond to SNP density.

**Additional file 2: Table S1.** The clean data details of resequencing samples. **Table S2.** Mapping details of resequencing samples. **Table S3.** Distribution of SNPs after LD pruned. **Table S4.** Estimates of genetic correlations between clutch traits g using the relationship matrix that blends genomic information. **Table S5.** GWAS significant SNPs. **Table S6.** Annotations of GWAS significant SNPs. **Table S7.** The relationship between SNP genotypes with phenotypes. **Table S8.** The results of KEGG analysis. **Table S9.** The results of GO analysis.

## Data Availability

The genome sequence data reported in this article is being uploaded to the genome sequence file of the BIG Data Center of the Beijing Institute of Genomics, Chinese Academy of Sciences, and is publicly available from http://bigd.big.ac.cn (CRA007364).

## Abbreviations

AFE: Age at first egg
BWFE: Body weight at first egg
EMMAX: Efficient mixed-model association expedited
FWLCS: The first week when the longest clutch starts
GRM: Genetic relationship matrix
GWAS: Genome-wide association study
LC: Longest clutch until 52 week of age
LNDWEL: The longest number of days without egg-laying until 52 week of age
LR: Laying rate
LWLCS: The last week of longest clutch ends
MLM: Mixed linear model
NELL2: Neural EGFL like 2
NUMC: Number of clutches
PCA: Principal component analysis
PCR: Polymerase chain reaction
PLCL1: Phospholipase D1
REML: Restricted maximum likelihood
SMYD3: SET and MYND domain containing 3
SMYD9: SET and MYND domain containing 9
SNP: Single nucleotide polymorphism
SPTLC2: Serine palmitoyltransferase long chain base subunit 2
