# Supplementary material for: Elucidation of the genetic determination of clutch traits in Chinese local chickens of the Laiwu Black breed
Source: BMC Genomics. 2023 Nov 16;24:686. doi: 10.1186/s12864-023-09798-0 (PMC10652520; doi:10.1186/s12864-023-09798-0)
Supplement: Supplementary file 1 — Additional file 1: Figure S1. SNP density plot across the 30 chromosomes of chicken showing the number of SNPs within a 1-Mb window size. The horizontal axis represents the chromosome length in Mb. Different colors correspond to SNP density. Figure S2. Principal component analysis (PCA). Figure S3. Q-Q plot derived from GWASs for clutch traits. The Q-Q plot contains expected -log10-transformed P-values plotted against the observed -log10-transformed P-values. Figure S4. Manhattan plot derived from GWASs for the LC. Each dot on this figure corresponds to a SNP within the dataset, and the horizontal red and blue lines denote the genome-wide significance (3.42E-8) and suggestive significance thresholds (6.84E-7), respectively. The Manhattan plot contains -log10 observed P-values for genome-wide SNPs (y-axis) plotted against their corresponding position on each chromosome (x-axis). The horizontal axis represents the chromosome length in Mb. Different colors correspond to SNP density. Figure S5. Manhattan plot derived from GWASs for the FWLCS. Each dot on this figure corresponds to a SNP within the dataset, and the horizontal red and blue lines denote the genome-wide significance (3.42E-8) and suggestive significance thresholds (6.84E-7), respectively. The Manhattan plot contains -log10 observed P-values for genome-wide SNPs (y-axis) plotted against their corresponding position on each chromosome (x-axis). The horizontal axis represents the chromosome length in Mb. Different colors correspond to SNP density. Figure S6. Manhattan plot derived from GWASs for the LWLCE. Each dot on this figure corresponds to a SNP within the dataset, and the horizontal red and blue lines denote the genome-wide significance (3.42E-8) and suggestive significance thresholds (6.84E-7), respectively. The Manhattan plot contains -log10 observed P-values for genome-wide SNPs (y-axis) plotted against their corresponding position on each chromosome (x-axis). The horizontal axis represents the chromo [file 12864_2023_9798_MOESM1_ESM.docx]

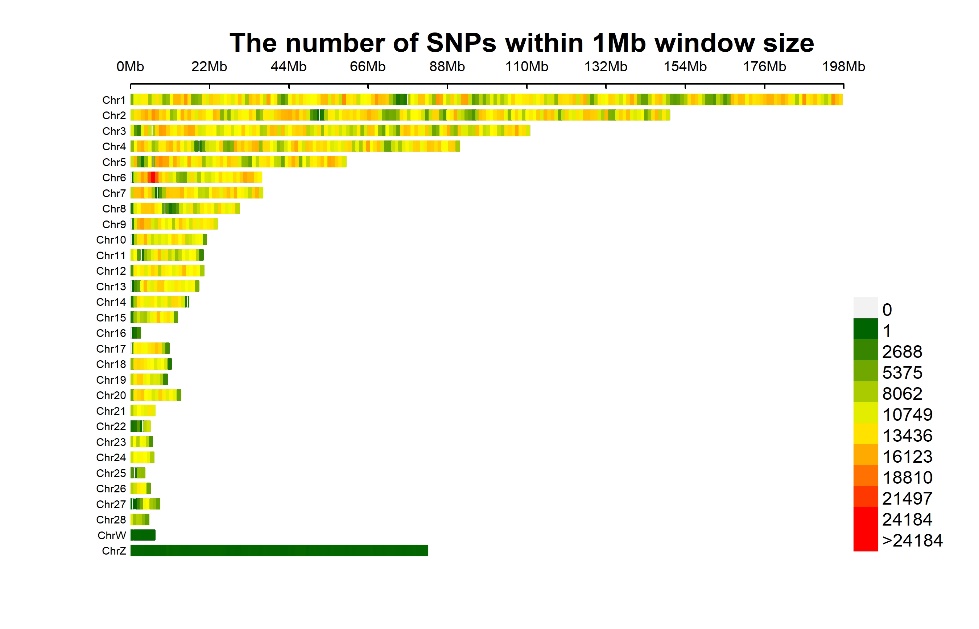


Figure S1. SNP density plot across the 30 chromosomes of chicken showing the number of SNPs within a 1-Mb window size. The horizontal axis represents the chromosome length in Mb. Different colors correspond to SNP density.


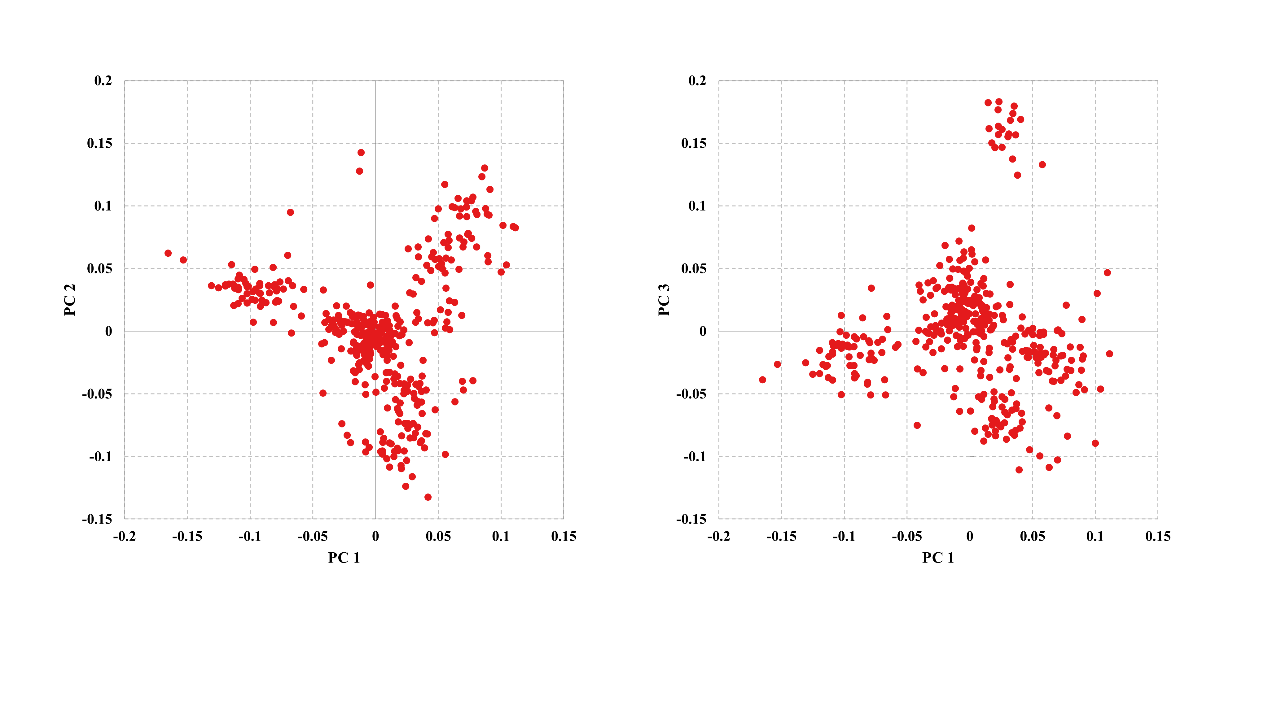


Figure S2. Principal component analysis (PCA)


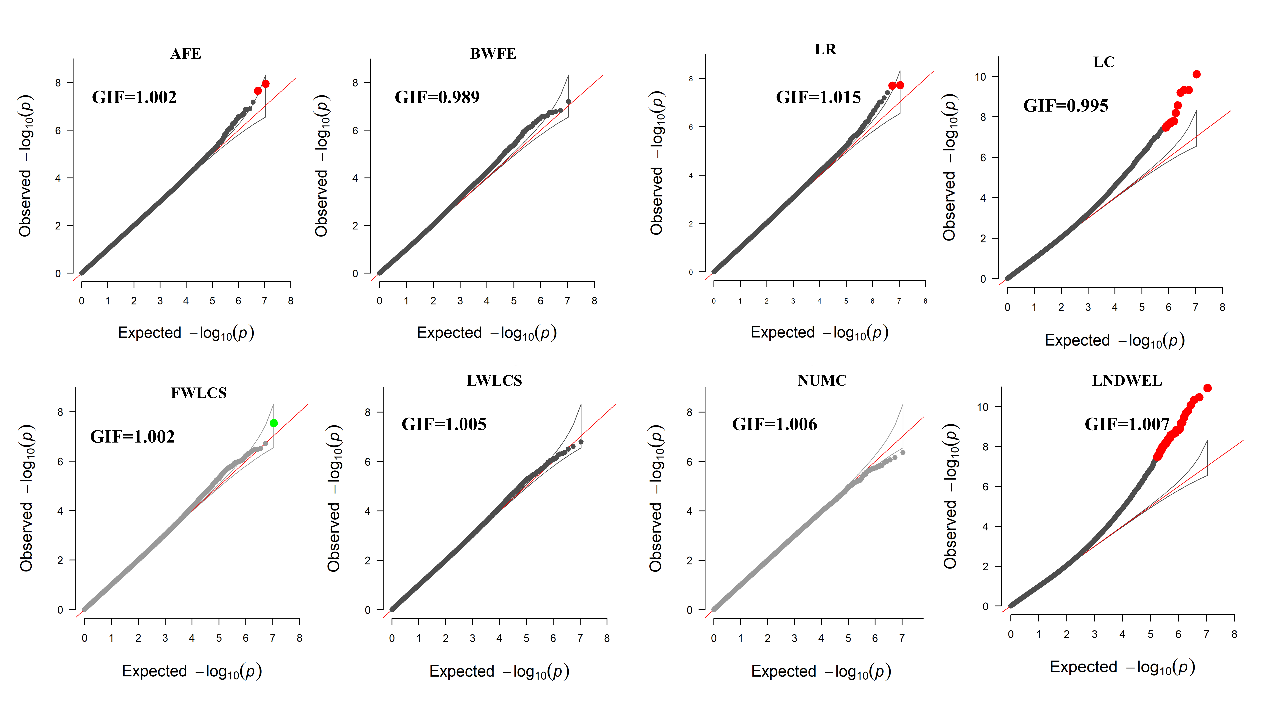


Figure S3. Q-Q plot derived from GWASs for clutch traits. The Q-Q plot contains expected -log10-transformed P-values plotted against the observed -log10-transformed P-values.


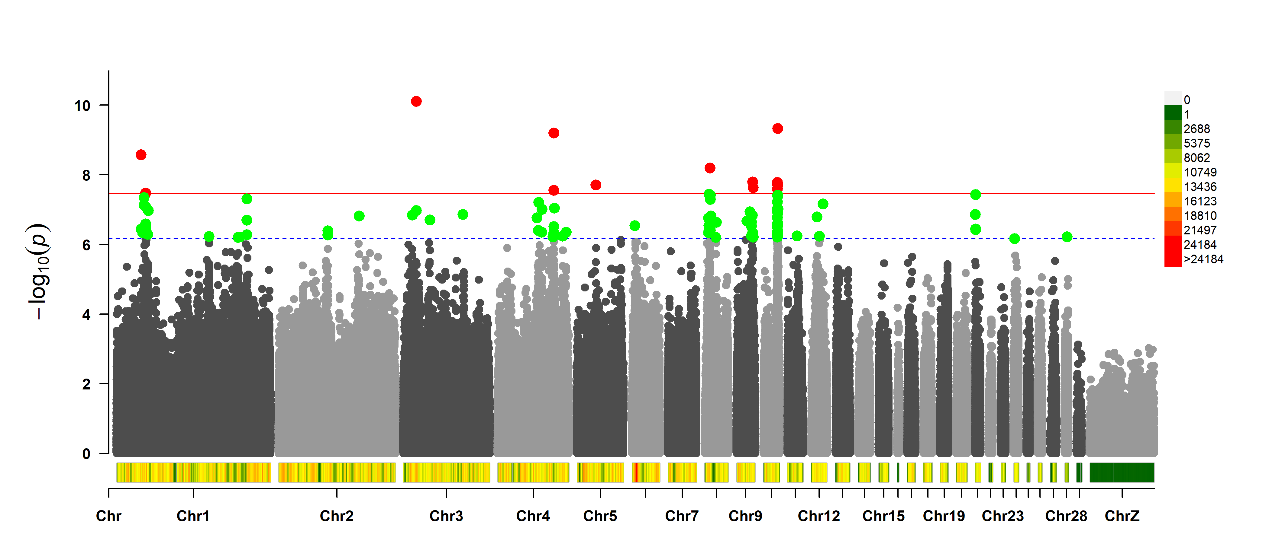


Figure S4. Manhattan plot derived from GWASs for the LC. Each dot on this figure corresponds to a SNP within the dataset, and the horizontal red and blue lines denote the genome-wide significance (3.42E-8) and suggestive significance thresholds (6.84E-7), respectively. The Manhattan plot contains -log10 observed P-values for genome-wide SNPs (y-axis) plotted against their corresponding position on each chromosome (x-axis). The horizontal axis represents the chromosome length in Mb. Different colors correspond to SNP density.


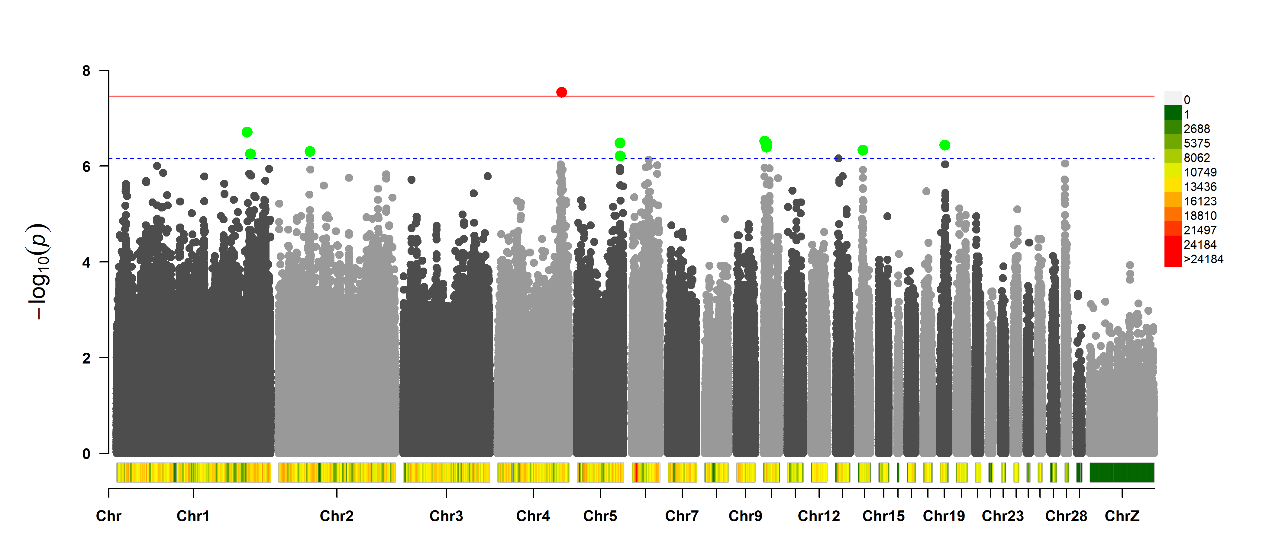


Figure S5. Manhattan plot derived from GWASs for the FWLCS. Each dot on this figure corresponds to a SNP within the dataset, and the horizontal red and blue lines denote the genome-wide significance (3.42E-8) and suggestive significance thresholds (6.84E-7), respectively. The Manhattan plot contains -log10 observed P-values for genome-wide SNPs (y-axis) plotted against their corresponding position on each chromosome (x-axis). The horizontal axis represents the chromosome length in Mb. Different colors correspond to SNP density.


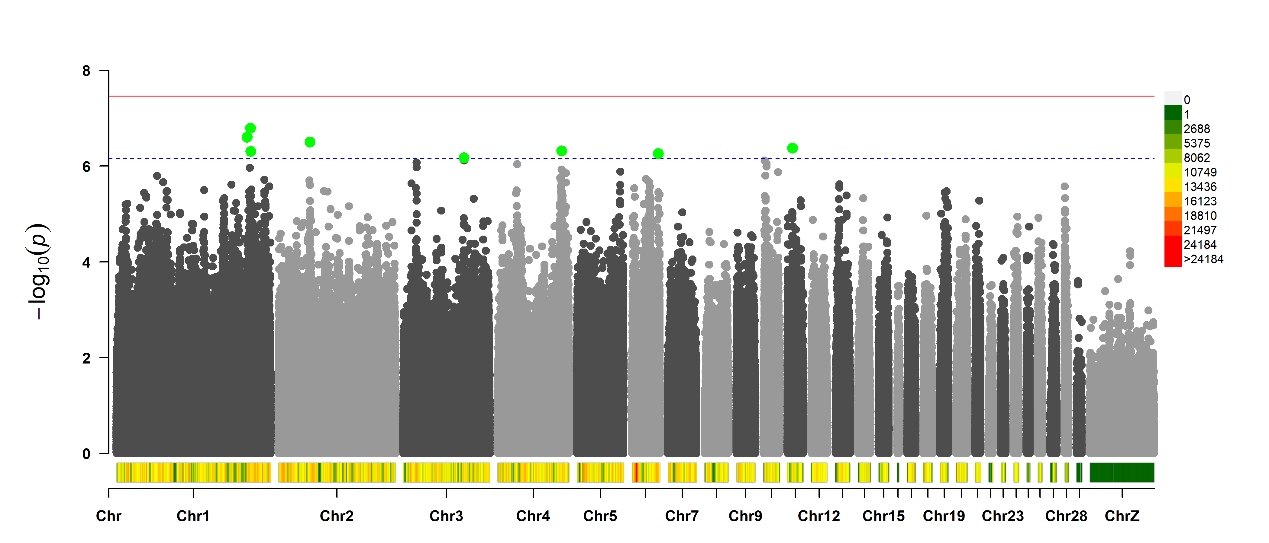


Figure S6. Manhattan plot derived from GWASs for the LWLCE. Each dot on this figure corresponds to a SNP within the dataset, and the horizontal red and blue lines denote the genome-wide significance (3.42E-8) and suggestive significance thresholds (6.84E-7), respectively. The Manhattan plot contains -log10 observed P-values for genome-wide SNPs (y-axis) plotted against their corresponding position on each chromosome (x-axis). The horizontal axis represents the chromosome length in Mb. Different colors correspond to SNP density.


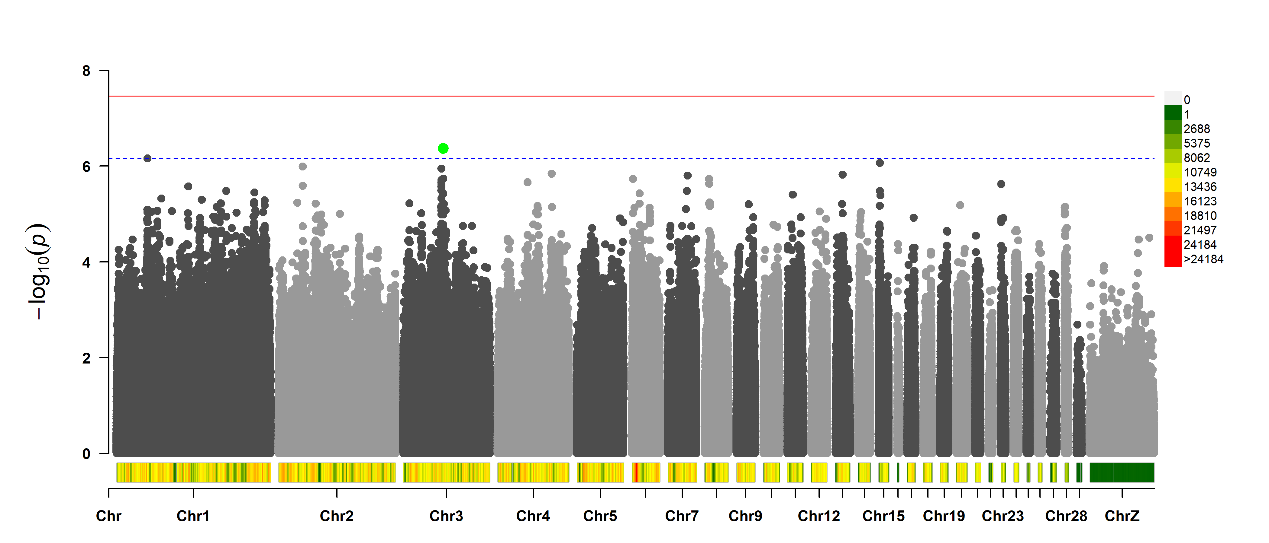


Figure S7. Manhattan plot derived from GWASs for the NUMC. Each dot on this figure corresponds to a SNP within the dataset, and the horizontal red and blue lines denote the genome-wide significance (3.42E-8) and suggestive significance thresholds (6.84E-7), respectively. The Manhattan plot contains -log10 observed P-values for genome-wide SNPs (y-axis) plotted against their corresponding position on each chromosome (x-axis). The horizontal axis represents the chromosome length in Mb. Different colors correspond to SNP density.


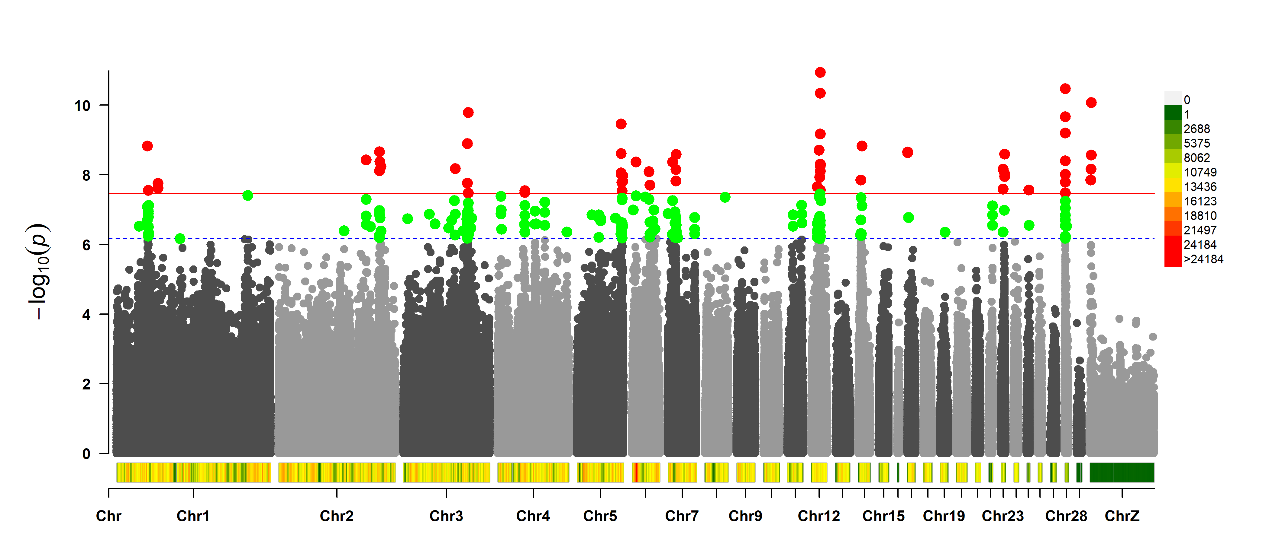


Figure S8. Manhattan plot derived from GWASs for the LNDWEL. Each dot on this figure corresponds to a SNP within the dataset, and the horizontal red and blue lines denote the genome-wide significance (3.42E-8) and suggestive significance thresholds (6.84E-7), respectively. The Manhattan plot contains -log10 observed P-values for genome-wide SNPs (y-axis) plotted against their corresponding position on each chromosome (x-axis). The horizontal axis represents the chromosome length in Mb. Different colors correspond to SNP density.
